# Transparent Low Molecular Weight Poly(Ethylene Glycol) Diacrylate-Based Hydrogels as Film Media for Photoswitchable Drugs

**DOI:** 10.3390/polym9120639

**Published:** 2017-11-23

**Authors:** Théophile Pelras, Sarah Glass, Tom Scherzer, Christian Elsner, Agnes Schulze, Bernd Abel

**Affiliations:** 1 Leibniz-Institute of Surface Modification, Permoserstraβe 15, 04318 Leipzig, Germany; tpel6241@uni.sydney.edu.au (T.P.); sarah.glass@iom-leipzig.de (S.G.); tom.scherzer@iom-leipzig.de (T.S.); christian.elsner@iom-leipzig.de (C.E.); bernd.abel@iom-leipzig.de (B.A.); 2 Key Center for Polymers and Colloids, School of Chemistry, University of Sydney, Sydney, NSW 2006, Australia

**Keywords:** hydrogels, drug release, photopolymerization, photosensitizer, poly(ethylene glycol) diacrylate, photodynamic therapy

## Abstract

Hydrogels have shown a great potential as materials for drug delivery systems thanks to their usually excellent bio-compatibility and their ability to trap water-soluble organic molecules in a porous network. In this study, poly(ethylene glycol)-based hydrogels containing a model dye were synthesized by ultraviolet (UV-A) photopolymerization of low-molecular weight macro-monomers and the material properties (dye release ability, transparency, morphology, and polymerization kinetics) were studied. Real-time infrared measurements revealed that the photopolymerization of the materials was strongly limited when the dye was added to the uncured formulation. Consequently, the procedure was adapted to allow for the formation of sufficiently cured gels that are able to capture and later on to release dye molecules in phosphate-buffered saline solution within a few hours. Due to the transparency of the materials in the 400–800 nm range, the hydrogels are suitable for the loading and excitation of photoactive molecules. These can be uptaken by and released from the polymer matrix. Therefore, such materials may find applications as cheap and tailored materials in photodynamic therapy (i.e., light-induced treatment of skin infections by bacteria, fungi, and viruses using photoactive drugs).

## 1. Introduction

Hydrogels can be simply defined as three-dimensional networks made of hydrophilic polymer chains that are able to retain a large amount of water or biological fluids in their swollen state [1,2,3,4]. Such networks can be either chemically (i.e., via covalent bonds) or physically (i.e., via entanglements, hydrogen bonds, crystallites, or van der Waals interactions) crosslinked, depending on the desired properties [2,4]. Their ability to absorb water arises from a hydrophilic polymer network, which can provide diffusion rates for water, nutriments, oxygen, and cellular wastes that are close to those of biological tissues, resulting in an excellent bio-compatibility [2,3,5]. Therefore, hydrogels offer great potential as supporting materials for medical treatments, including among others contact lenses [6], artificial bones and cartilages [7], tissue engineering [8], cellular immobilization [9], separation of molecules and cells [10], in situ drug delivery materials [11], and bandages [12]. In this context, transparent hydrogels that are able to release photoactive molecules into the skin may be very interesting for numerous medical applications, including active bandages for photodynamic therapy at the skin surface. 

In photodynamic therapy, so-called photosensitizers are used as drugs. These photosensitizers can absorb light and transfer its energy to molecular oxygen. Thereby, the natural triplet oxygen is converted into reactive oxygen species (ROS), such as singlet oxygen, superoxide radicals, or hydroxyl radicals [13,14,15]. These ROS are known to be cytotoxic and have been used in cancer therapy since the 1970s [16]. Furthermore, they have been used in antimicrobial photodynamic therapy and in dental medicine for several years [17,18].

Over the past years, hydrogels have been formed with a broad spectrum of building blocks and through a large range of methods. Building blocks, including simple polymer chains like poly(ethylene glycol) (PEG) [19], natural polymer chains such as chitosan/dextran [20,21], and gelatin [22,23], or from polymers that exhibit peculiar properties like poly(*N*-isopropylacrylamide) (PNIPAm) [24,25], which undergoes a temperature-driven physical change [26,27]. Despite these breakthroughs in polymer engineering, PEG remains a first choice building block to form bio-compatible polymer materials for medical applications. Besides the great affinity between the polymer chains and water molecules, PEG offers a large catalog of precursors, including (meth)acrylate-functionalized PEG, among others. Due to its acrylic functions located at both ends of the chain, poly(ethylene glycol) diacrylate (PEGDA) can be easily polymerized using either photoinitiators [28], thermal initiators [29], or electron beam irradiation [19]. 

Numerous techniques have also been explored for the formation of hydrogels, such as the already mentioned photo- or thermal polymerizations and electron-beam crosslinking of solutions, the electron beam crosslinking of (meth)acrylates in a frozen solution state (therefore named cryogels) [30], or the photopolymerization of PNIPAm [31] or photoactive resins [32] in an evaporative solvent environment. Despite the numerous methods mentioned above, we believe that the photopolymerization of PEG building blocks in water-based media is one of the best alternatives due to the availability and affordability of the raw materials, the environment-friendly process, and the ease of the setup.

Several research groups have already studied photo-crosslinkable PEG-based hydrogels with the aim to use them in medical applications. For instance, Schmidt et al. [27,33,34] thoroughly studied PEG hydrogels that were reinforced with silica and hydroxyapatite nanoparticles for cartilage replacement. The outcome of their work showed that the addition of the particles leads to the large enhancement of the mechanical properties of the material, but also decreased its swelling ability and transparency.

Other groups, like the one of Marchant [35], studied the effects of the ratio between monoacrylated and diacrylated PEG chains on the final properties of the materials that are formed by UV-A curing. Their experiments mostly confirmed the assumption that a higher bifunctional vs. monofunctional PEG ratio increases the density of the network and improves its mechanical properties, but decreases the swelling ability. Moreover, it was shown that an increase of the total concentration of the acrylated polymer chains in solution lowers the swelling ratio of the final material.

Sah and coworkers [7] studied the effect of PEGDA molecular weight (in the 500–10,000 g·mol^−1^ range) and the concentration in solution on the properties of UV-A-cured cartilage-like materials. As expected, both molecular weight and concentration have a strong influence on the mechanical and swelling properties of the final objects. For instance, the swelling ratio of the materials was increased by rising the molecular weight and decreasing the concentration of the monomer, whereas the compressive and tensile moduli were improved by increasing the solution concentration from 10 to 40 wt %.

Other groups, like Göpferich et al. [36], studied the properties of hydrogels based on four-armed PEG as materials for drug delivery. In order to follow the release kinetics, fluorescent-labelled dextrans of various molecular weights were introduced into the gel, and fluorescence recovery after photobleaching experiments were studied and compared with theoretical values. Whereas, the polymer concentration in solution—up to 10%—led to a slight change of the kinetics only, the molecular weight of the fluorescent compounds had a tremendous effect and large molecules had a much lower diffusion coefficient than the small ones.

Finally, Anseth et al. [37] have explored the use of lithium acylphosphinate salt as photoinitiators for the encapsulation of living cells by PEGDA. Their custom-made photo-initiating system has shown better solubility when compared to commercially available systems and better absorption properties, enabling the curing of the network using 365 or 405 nm lamps with good efficiencies and 95% cell survivability. 

Most of the recent work observed high molecular weight PEGDA, usually in the 6000–30,000 g·mol^−1^ range. Transparent gels from low molecular weight PEGDA were not considered to have interesting features with respect to molecular carriers with a sufficient loose molecular network yet. However, such materials may find applications as external drug delivery carriers (i.e., applied on the skin) and play a role as “active bandages” that are able to disinfect wounds. Although the transparency is not compulsory for “conventional” bandages, it can be one of the key characteristics of materials for the delivery of porphyrin- or tetrapyrrole-like photoactive drugs into the skin, like, for instance, in photoacoustic imaging [38] and photodynamic therapy [39,40]. Moreover, the development of new water-soluble tetrapyrrole-type photosensitizers with excellent absorption toward the upper part of the visible spectrum [15] is pushing forward the photodynamic therapy and thereby the need of easy-to-make and affordable drug-carrying materials. 

In this study, hydrogel materials were prepared by UV-A photopolymerization of low molecular weight PEGDA chains using a commercially available water-soluble. The aim of this work was to demonstrate the formation of inexpensive, easy-to-make, transparent hydrogel materials that are capable of releasing absorbed large organic molecules, like, for instance, tetrapyrrole-like structures, which are commonly used in photodynamic therapy. The morphology, the molecular release, as well as the polymerization characteristics were investigated, and the materials were characterized with respect to potential applications as a transparent photo-drug carrier. 

## 2. Materials and Methods 

### 2.1. Materials

**Figure 1:**
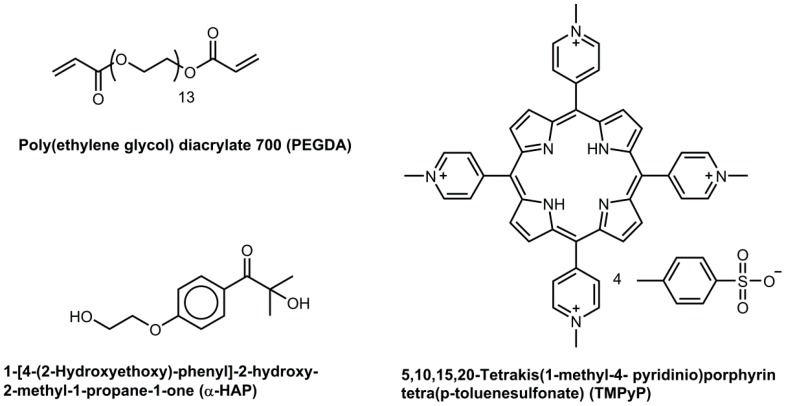
Molecular structures of the chemicals used during the study.

Poly(ethylene glycol) diacrylate with an average molecular weight of 700 g·mol^−1^ (PEGDA), phosphate buffered saline solution (PBS; pH = 7.4) and the tetrapyrrole dye (5,10,15,20-tetrakis(1-methyl-4-pyridinio)porphyrin tetra(*p*-toluenesulfonate); TMPyP), were purchased from Sigma-Aldrich (Saint-Louis, MO, USA). The water-soluble photoinitiator 1-[4-(2-hydroxyethoxy)-phenyl]-2-hydroxy-2-methyl-1-propane-1-one (α-HAP) was bought from BASF (Ludwigshafen, Germany). The molecules used are represented in Figure 1. All of the chemicals were used as received.

### 2.2. Formulations

PEGDA was given into PBS at 30 wt %, and α-HAP was added at either 0.05, 0.1 or 0.5 wt %. In some cases, TMPyP was added to the formulations at a concentration of 0.01 wt %. The mixtures were sonicated for 20 min to allow proper dissolution of each component. These polymerizable mixtures will be called “0.05 wt %”, “0.1 wt %” and “0.5 wt %”, respectively, throughout this paper.

### 2.3. Real-Time Infrared Spectroscopy

The real-time infrared analyses were adapted from previous investigations on the photopolymerization of various acrylic systems [41,42]. Measurements were performed using a Varian 670 Fourier-transform infrared (FTIR) spectrometer from Varian (now Agilent, Santa Clara, CA, USA) equipped with a Golden Gate diamond attenuated total reflectance (ATR) accessory from Specac (Orpington, UK). Infrared measurements were recorded in the 4000–600 cm^−1^ spectral range with 4 cm^−1^ spectral and 0.242 s time resolution using a MCT detector. Kinetic conversion profiles were calculated according to Formula (1) (with *A_t_* the area of the acrylate band at 1410 cm^−1^ at any time and *A_t_*_=0_ the band area at 1410 cm^−1^ before curing) using the decay of the absorption of the =CH_2_ deformation band at 1410 cm^−1^. Even if other acrylic bands (for instance, the C=C stretching vibration at 1636 cm^−1^ or the =CH_2_ twist at 811 cm^−1^) [43] are commonly used to monitor the photopolymerization kinetics, the strong signal of the solvent prevented an accurate measurement using the 1636 and 811 cm^−1^ bands.

(1) $$\%\;conv.=100\times \frac{A_{t}}{A_{t=0}}$$

The induction time (i.e., the time before the reaction starts), the final conversion, and the maximal reaction rate are the main parameters of the photopolymerization kinetics. Whereas, the two first parameters can be directly determined from the plot, the reaction rate *R_p_* at any time was calculated according to the Formula (2) (with [*M*] the monomer concentration).

(2) $$R_{p}=-\frac{d[M]}{dt}$$

The irradiation of the formulations was performed with an UV-A lamp PL-S 9W/2P BLB from Philips (Eindhoven, The Netherlands) mounted in a home-made irradiation box, which was adapted to the ATR unit (see Figure S1) for real-time infrared investigations. The UV lamp emitted light between 340 and 410 nm with a maximal intensity at 365 nm. The light intensity received by the sample at a distance of 1.5 cm was 12 mW·cm^−2^, which was determined with a small UV radiometer from Epigap (now Jenoptik Polymer Systems, Berlin, Germany). The lamp was switched on for 5 min before the beginning of the synthesis to reach a maximal and stable intensity. A manual shutter was used to start the polymerization.

To achieve a reproducible thickness, one drop of formulation was applied on the ATR crystal and was covered by a 6 mm thick quartz plate with a gap of 4.5 μm in its lower side (93% of light transmitted through the quartz plate), which was made by ion beam etching [41]. In order to cure the formulation only on the ATR crystal (and hence to facilitate the release of the quartz plate after the end of the experiment), the top side of the quartz plate was metalized with aluminum. A hole with a diameter of 5 mm was kept clear as a window for the incident UV radiation. Additionally, the aluminum layer was blackened for safety reason. In several cases, inertization was performed by flushing the irradiation box with nitrogen for two minutes before the start of the irradiation and the gas flow was kept during the whole measurement.

### 2.4. UV-Vis Spectroscopy

UV-Vis absorbance spectra of the chemicals were recorded in Milli-Q water at concentrations of 10^−3^ mol·L^−1^ for PEGDA, 10^−5^ mol·L^−1^ for α-HAP, and 2.5 × 10^−6^ mol·L^−1^ for the tetrapyrrole. A Cary 5000 UV-Vis spectrometer from Agilent (Santa Clara, CA, USA) with a spectral range of 200–800 nm and 1 nm resolution was used. Extinction coefficients were calculated using the Beer-Lambert law.

For the drug release experiments, formulations were prepared, cast into 1 mm deep molds, and polymerized for 20 min the UV-A lamp. They were then freeze-dried at −60 °C and 50 mbar for a day and were placed into a 0.01 wt % TMPyP solution in PBS for 12 h. The dry samples swelled and absorbed dye molecules during this process. The excess of solvent was carefully removed from the samples using a dust-free wipe and they were individually placed into glass bottles containing 25 mL of PBS to simulate contact with human tissues. Masses of dried and swollen samples and solvent were determined for further calculations and data normalization. UV-Vis spectra of the solvent were then recorded within a spectral range of 200–800 nm and at 1 nm resolution after 0.25, 0.5, 1, 2, 4, 6, 8, 12, and 24 h using the above-mentioned spectrometer. 

An UV-2101PC UV-Vis spectrometer from Shimadzu (Kyoto, Japan) was used to record transmittance curves of the hydrogels made from the same formulations using the mold-casting method. The studied range was set to 200–800 nm with 0.5 nm resolution. Samples had an average thickness of 1 mm. 

### 2.5. Scanning Electron Microscopy

The hydrogels for SEM images and transparency studies were made of the same formulations, which were cast into 1 mm deep molds and were irradiated with a dose of 2500 mJ·cm^−2^ using a medium-pressure mercury lamp. The synthesized samples were washed in PBS for 24 h and in deionized water for 2 h before being dried at room temperature and atmospheric pressure for 24 h. After sputter-deposition of a chrome layer, scanning electron microscopy (SEM) was performed using an Ultra-55 microscope equipped with a Gemini Detector (both from Zeiss, Jena, Germany) at a pressure of 2 × 10^−5^ mbar and 5 kV voltage.

### 2.6. Detection of Unreacted Products

Despite a relatively long exposure to UV radiation, not all of the chemicals involved in the hydrogel network formation may have reacted. In order to determine if unreacted chemicals can escape, hydrogel samples were prepared, as mentioned above, and were washed in PBS for several days with a complete change of the PBS every 24 h. These washing solutions were analyzed by gas-chromatography/mass-spectrometry (GC-MS) by mixing 2 mL of washing solution with 5 µL ethanol, which was used as internal standard. To compare the amount of residual substances the relative amount was calculated according to Formula (3)

(3) $$rel.\;amount=\frac{A_{t}}{A_{24h}}$$

where *A_t_* is the normalized area of the peak in the chromatogram at the time *t* and *A*_24h_ is the standardized area of the peak in the chromatogram at 24 h. As inlet, a SPME (solid phase microextraction) was used. The gas chromatography measurements were performed on an Agilent Technologies 6890N network GC system with Agilent 5973 network mass selective detector (Santa Clara, CA, USA), quadrupole mass analyser, pressure: 2.3 × 10^−5^ torr) equipped with a multipurpose sampler MPS2. The column was a HP-5MS with 0.25 mm diameter and 30 m length.

### 2.7. Swelling Ratio

Freshly synthesized hydrogels were stored in PBS for 24 h and water for 2 h to determine the mass of the wet hydrogel (*m_wet_*). After 24 h of drying at room temperature and under atmospheric pressure the mass of the dry hydrogel (*m_dry_*) was determined. The swelling ratio *Q* was calculated according to Formula (4):

(4) $$Q=\frac{m_{wet}}{m_{dry}}$$

## 3. Results & Discussion

### 3.1. UV-Vis Spectroscopy

**Figure 2:**
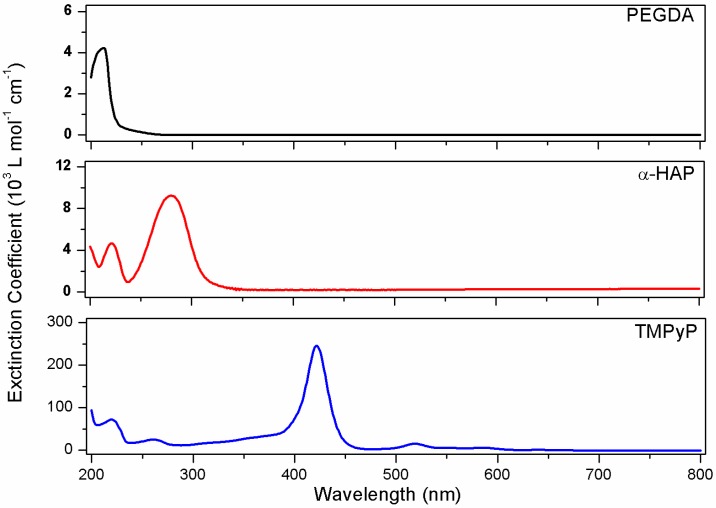
Extinction coefficients of poly(ethylene glycol) diacrylate (PEGDA) (10^−3^ mol·L^−1^), α-HAP (10^−5^ mol·L^−1^), and TMPyP (2.5 × 10^−6^ mol·L^−1^) in deionized water.

The efficiency of UV curing strongly depends on the absorption properties of the involved compounds and the spectral emittance characteristics of the irradiation system. Spectral overlap of the photon emittance and the absorbance band of the photoinitiator is an essential requirement to trigger free-radical polymerization of acrylic monomers via radical generation by the decomposition of the photoinitiator. The situation becomes more complicated if electron-rich molecules are present in the reaction mixture, because they can absorb a significant amount of photons in the absorption range of the photoinitiator. Thus, the first step of this work was (1) to determine the UV-Vis absorption properties of the various compounds in the formulation, (2) to investigate the efficiency of the photoinitiator at 365 nm, and (3) to compare its absorption properties to that of the other components of the formulation. The resulting data are displayed in Figure 2.

The various chemicals have characteristic absorption bands in the 200–800 nm spectral range. PEGDA has a very weak absorption spectrum due to its linear structure based on σ-bonds and the absence of chromophoric groups. Its highest extinction coefficient is reached at 213 nm with 4200 ± 200 L·mol^−1^·cm^−1^. The photoinitiator has a characteristic absorption band at 280 nm with an extinction coefficient of 9200 ± 300 L·mol^−1^·cm^−1^, as well as a weaker band at 221 nm. Finally, TMPyP has a complex absorption spectrum that is characterized by a very intense Soret absorption band at 422 nm with 246,000 ± 5000 L·mol^−1^·cm^−1^, several less intense bands below 200 nm, at 219 nm, and at 261 nm, and several Q bands between 475 and 675 nm. 

The main absorption band of the photoinitiator clearly does not fit perfectly with the emission spectrum of the UV-A lamp (main emission band at 365 nm). Very few commercially available photoinitiators are water-soluble and they generally do not have absorption spectra that match the emission of typical UV-Vis lamps. Nonetheless, the extinction coefficient of this chemical at 365 nm, measured to be 200 ± 100 L·mol^−1^·cm^−1^, still allows for the formation of free-radical species and thus the formation of a PEG-based crosslinked network [33,35]. The extinction coefficient of PEGDA at 365 nm was measured to be 5 L·mol^−1^·cm^−1^. So, the absorption of the monomer does not interfere at all with that of the photoinitiator. However, TMPyP has an extinction coefficient of 32,000 ± 1000 L·mol^−1^·cm^−1^ at 365 nm, so a strong reduction of the photopolymerization efficiency was expected in dye-containing formulations.

### 3.2. Investigation of the Hydrogel Photopolymerization 

Real-time FTIR spectroscopy was used to monitor the photopolymerization kinetics and to determine the influences of the concentrations of α-HAP and dye, as well as the nitrogen inertization on induction time, maximum rate of reaction, as well as final conversion. During photopolymerization, free-radical species were formed and reacted with the PEGDA acrylic double bonds, inducing a reduction of the intensity of the band assigned to the =CH_2_ deformation vibration (see Figure S2). The decrease of this signal was used to calculate the conversion of the monomer, while the other bands remained the same, proving that only the acrylic functions are involved in the reaction pathway. 

**Figure 3:**
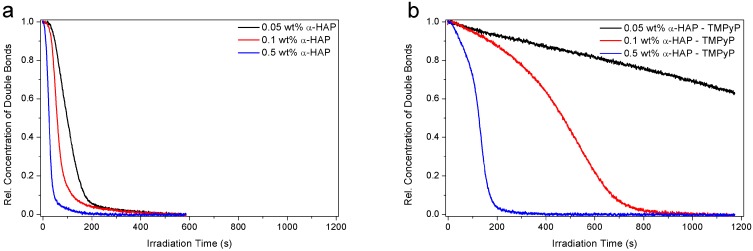
Photopolymerization kinetics of hydrogel formulations using three different α-HAP concentrations under atmospheric conditions and light intensity of 12 mW·cm^−2^: (**a**) without dye and (**b**) with TMPyP at 0.01 wt %.

The first set of samples studied did not contain any dye and the measurements were performed under atmospheric conditions. The results presented in Figure 3a clearly show that the concentration of α-HAP has a significant influence on the reaction kinetics. Indeed, a lower induction time and a higher reaction rate were achieved when the weight percentage of photoinitiator was increased. After 300 s, almost 100% of the double bonds disappeared in all of the samples, so a fully-cured network was achieved after this time using only 0.05 wt % of photoinitiator. A higher concentration only enhances the maximal rate of the reaction, from 0.008 s^−1^ for the lowest photoinitiator concentration to 0.033 s^−1^ for the highest one (i.e., a 400% increase). 

Then, a second series of measurements was performed using dye-containing formulations with a tetrapyrrole concentration of 0.01 wt %. The results in Figure 3b prove that even a very small weight percentage of dye in the formulation tremendously decreased the photopolymerization efficiency. A drastic drop of the maximal reaction rate was observed, from 0.008 to 0.0004 s^−1^, for the lowest photoinitiator concentration (i.e., a twentieth of the speed) and 0.033 to 0.012 s^−1^ for the highest concentration (i.e., a third of the rate). This effect was obviously even stronger when the photoinitiator concentration was low, leading to very slow double bonds conversion and non-fully cured materials.

Finally, all of the formulations were studied under inert atmosphere by flushing the irradiation box with nitrogen (see Figure S3). Molecular oxygen is highly reactive and is able to scavenge free-radical species and strongly reduces the reaction efficiency [44,45]. Moreover, oxygen is known to dissolve in water at a concentration of 8.3 mg L^−1^ [46,47,48], where it can scavenge free-radicals and stop the network from growing within the water-based formulation. Many solutions have already been studied to reduce oxygen inhibition in free-radical polymerizations [44] (like, for instance, using special additives [49], more efficient photoinitiating systems [50], or higher light intensity), but the most effective approach appears to be inertization. The removal of oxygen in dye-free formulations (Figure S3a) allowed for a slight enhancement of the reaction rate, but similar final conversions were measured. The use of nitrogen during the polymerization of dye-containing formulations (Figure S3b) led however to more significant improvements, especially in the case of low photoinitiator concentration. Unfortunately, the use of an inert atmosphere during the photopolymerization appears not to be efficient enough to counterbalance the negative effects that are induced by the presence of the dye. These enhancements are not high enough to allow for the curing of 1 mm thick materials within 20 min. An increase of the photoinitiator concentration could overcome this problem, but resulted in a too stiff and brittle network. Due to these considerations, hydrogels were synthesized from mixtures of PEGDA, α-HAP, and water only, and the dye was trapped in the network later on. All of the kinetics data are displayed in Table S1 in the Supporting Information.

### 3.3. Transparency

**Figure 4:**
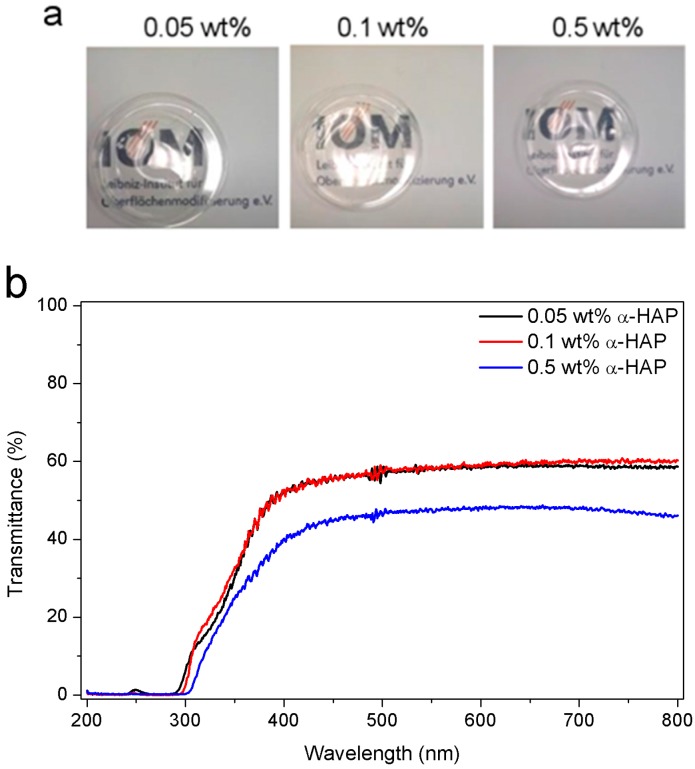
Photographs of the final materials (**a**) and transparency measurements of the samples (**b**) made with three different photoinitiator concentrations: 0.05 wt %, 0.1 wt %, and 0.5 wt %.

Clear and transparent materials may find applications as release media for photoactive drugs. Therefore, a high transmission of the light in the 400–800 nm range is essential. Transparency measurements, as well as photographs of 1 mm thick samples are presented in Figure 4. The samples are perfectly translucent and have a slightly yellow color, which might be caused by remaining photoinitiator (Figure 4a). The high transmission at wavelengths higher than 400 nm is a strong advantage for the use of hydrogels as photoactive drug carriers since light at the main absorption band of TMPyP at 420 nm is not attenuated by the matrix. The transmittance of the sample made with 0.5 wt % α-HAP was not higher than 49%, whereas the samples produced with lower photoinitiator concentrations had similar transmission spectra with a maximal transmittance of 61% (Figure 4b). Therefore, we believe that the transmittance is mainly determined by the density of the acrylic mesh [51]. The transmittance for the hydrogels with the highest photoinitiator concentration is probably lowered because more photoinitiator is attached to the network and its structure is slightly changed.

### 3.4. Surface Morphology

**Figure 5:**
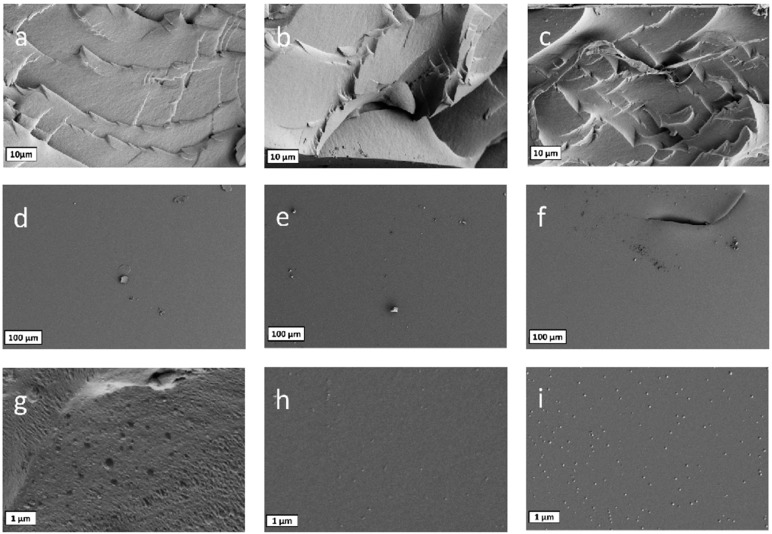
Scanning electron microscopy (SEM) images of cross sections (**a**–**c**) and surfaces (**d**–**i**) of the hydrogels materials with 0.05 wt % (**a**,**d**,**g**) 0.1 wt % (**b**,**e**,**h**) and 0.5 wt % (**c**,**f**,**i**) photoinitiator. White bars correspond to 10 µm (**a**–**c**), 100 µm (**d**–**f**), and 1 µm (**g**–**i**).

Scanning electron microscopy was used to investigate the surface of the samples, and to check for the presence or absence of pores in the structure. Size, number, as well as distribution of pores may have a strong influence on the physical properties of the material and on the trapping and release abilities for chemical compounds. SEM images are displayed in Figure 5. The surface of the hydrogels is very smooth, homogenous, and without any defects or holes. On enlargements (scale 1 µm, Figure 5g–i), small salt crystals of residual PBS appear. Neither on the surface nor on the cross sections, meshes or large pores can be detected due to the short PEG chains used for the build-up of the matrix. There is no difference in the morphology when comparing the hydrogels that had been synthesized with different amounts of photoinitiator, meaning that the photoinitiator concentration has no influence on the microscopic structure. 

### 3.5. Release Behavior

First, swelling ratios have been measured for the different formulations. No significant differences between the cast polymer discs were observed. The swelling ratios were found to be 3.43 ± 0.07 for hydrogels made with 0.05 wt % α-HAP, 3.48 ± 0.03 for 0.1 wt % and 3.54 ± 0.03 for 0.5 wt %. Consequently, the swelling behaviour is not influenced by the photoinitiator concentration for this system.

**Figure 6:**
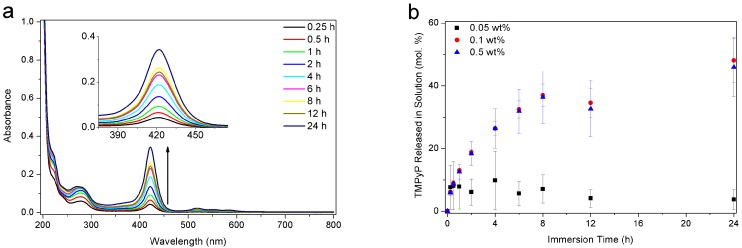
UV-Vis spectra of the phosphate buffered saline solution (PBS) solution various times after immersion of a TMPyP-loaded sample made with 0.5 wt % α-HAP (**a**) and plot of the molar percentages of TMPyP released into the PBS solution from dye-loaded samples made with the three different photoinitiator concentrations (**b**). The molar percentage of dye is calculated from the absorbed amount of dye in each hydrogel.

In order to study the dye release behavior of the hydrogels, cured samples were investigated after loading with TMPyP and immersion in PBS solution at 25 °C for the release. About 15 min after immersion, a slight yellow coloration of the solution appeared, which became more and more intense over a period of several hours. Figure 6a shows UV-Vis spectra of the solution after different time periods. The continuous increase of the band at 422 nm confirmed the release of TMPyP and its progressive diffusion out of the hydrogel. The concentrations of photoinitiator, and dye in the PBS solution were calculated from these UV-Vis spectra using the molar extinction coefficients of the compounds. The intake of model dye was calculated by the difference between the concentration of dye in the loading solution before and after immersion of the gels for 12 h. Hydrogels made from the highest and intermediate photoinitiator concentrations were measured to intake (6.7 ± 0.4) × 10^−8^ mol and (7.7 ± 0.6) × 10^−8^ mol of TMPyP, respectively. The gels formed from the lowest photoinitiator concentration could absorb (1.3 ± 0.1) × 10^−8^ mol of TMPyP only. The kinetics, as displayed in Figure 6b, show a rapid release of the TMPyP into the solution. Plateau concentrations were reached after 1 h for the gel made with 0.05 wt % α-HAP (5.0 × 10^−8^ mol·L^−1^, i.e., 8 mol %) and after more than 12 h for the other two samples (1.5 × 10^−6^ mol·L^−1^, i.e., 48 mol %, for 0.1 wt % and 1.2 × 10^6^ L·mol^−1^, i.e., 46 mol % for 0.5 wt %). Almost identical release curves were observed for samples that were made with 0.1 and 0.5 wt % α-HAP. The final molar percentages released were found to be about 50 mol %, whereas only 10 mol % were released from the sample prepared with 0.05 wt % α-HAP. Thus, samples that were synthesized with a low photoinitiator concentration (<0.05 wt %) seem not to form a network able to take up and release enough dye molecules, either via adsorption or trapping within the network. 

Two phenomena can explain why not all of the absorbed dye can escape the matrix: (1) some molecules are still physically trapped in the structure (via H-bonding for instance) like it was observed before e.g., for chloramphenicol in hydroxyethyl methacrylate hydrogels [52], and (2) the osmotic pressure does not allow for more dye to escape the hydrogel, which can be considered as an absorber. 

Due to the relatively large thickness of the samples, not all of the chemicals that are involved in the cross-linking process may have reacted and could potentially escape the network. A first insight of this phenomenon is given in Figure S4, which estimates the molar percentages of unreacted photoinitiator released into the PBS solution during the UV-Vis experiments. Despite the inclusion of the dye absorption into the calculations, due to its background absorption at the characteristic absorption peak of the α-HAP (i.e., 280 nm), this method lacks precision. Therefore, the hydrogels were immersed in PBS solution, which was changed every 24 h. These solutions were analyzed by SPME-GC-MS to identify the potentially formed photo-cleavage products and remaining unreacted monomer. 

**Figure 7:**
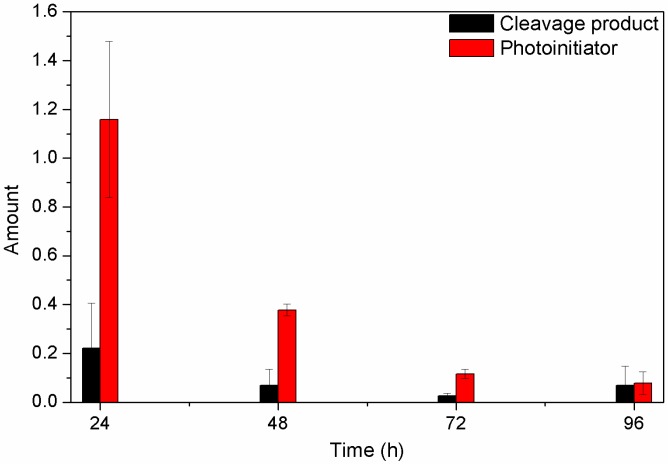
Chromatograms of photoinitiator and photo cleavage product in every washing solution taken after different time periods. The amount of residual compounds is related to the amount of the internal standard ethanol. Therefore, the area of the analyzed substances was divided by the peak area of ethanol.

The decreases of the residual compounds are displayed in Figure 7. The formulation with 0.1 wt % α-HAP was used because this formed the most promising gels. The photoinitiator, and its main photoproducts, as well as PEGDA, were identified by their *m*/*z* ratios (see Figures S5–S7). No signals fitting to PEGDA were found (Figure S8), meaning that the monomer was completely polymerized during the curing process.

The GC-MS data of the washing solutions demonstrate the excellent water solubility of the all-residual substances. We see a significant decrease of the amount of unreacted photoinitiator α-HAP and its decomposition product 4-(2-hydroxy)-ethoxybenzaldehyde within the first two washing steps. Even if it was not possible to determine the absolute amount of both substances by GC-MS, the amount of residual photoinitiator (and its cleavage product) must be lower than the initial concentration of 1 mg per 1 g gel. Nonetheless, the amounts of both the substances were reduced to less than 30% of the former amount after only one washing step (see Figure S9). This means that it is possible to wash out most of the photoinitiator residues and thus enhance the bio-compatibility of the system.

## 4. Conclusions

This study demonstrated the successful formation of low molecular weight PEGDA-based hydrogel networks via UV-A photopolymerization. Such materials offer the advantage to be cheap and easy-to-make when compared to more conventional systems that are based on high-molecular weight PEGDA monomers or electron-beam cross-linked materials. Therefore, they may be used as external drug delivery carriers for photodynamic therapy in the near future. From the three formulations that were studied, gels made with 0.1 or 0.5 wt % of α-HAP appeared to be the most suitable formulations. The resulting hydrogels have a smooth and homogenous surface with a very dense mesh. These meshes are in a molecular size. The photopolymerization of the gels by simultaneous incorporation of the drug (photosensitive dye) in the formulation mixture is difficult to achieve due to the high absorption of light by the dye. However, the inherent properties of the hydrogels easily allow for the absorption of the water-soluble drug subsequent to the gel preparation. The hydrogels were able to absorb the dye and to release almost half of the absorbed molecules within 24 h when immersed in phosphate buffer saline solution. GC-MS analyses have shown that all of the diacrylic monomers were bound in the polymer network and only traces of unreacted photoinitiator were left in the matrix. The photoinitiator and its photoproducts could be easily washed out by simple immersion of the gels into water. The enhancement of the network formation and biocompatibility tests have thus the highest priority for future work in order to form hydrogels that release an optimum percentage of dye and as little residual photoinitiator as possible. Furthermore, it is necessary to observe the behavior of further photo sensitizers. 

In summary, we report a functional and transparent hydrogel material, which can be employed as a reasonably flexible wound patch or patch for the treatment of skin irritation that is caused by fungi, viruses, or bacteria with light. The kinetics of drug loading and release enables an easy handling of the cheap ‘single use’ patch.

## Supplementary Materials

The following are available online at www.mdpi.com/2073-4360/9/12/639/s1. Figure S1: Scheme of the lamp and its irradiation box used for the photopolymerization of the mixtures (**a**). The scheme shows the irradiation box mounted on the ATR module of the RT-FTIR spectrometer. Detailed view of the sample deposited on the ATR crystal and calibrated using the quartz plate (**b**), Figure S2: Decrease of the IR band assigned to the =CH_2_ deformation vibration of the PEGDA at 1410 cm^−1^ in a hydrogel sample. The formulation containing 0.5 wt % α-HAP was photopolymerized in air with a light intensity of 12 mW cm^−2^ using the UV-A lamp, Figure S3: Photopolymerization kinetics of hydrogel formulations using three different α-HAP concentrations in dye-free formulations (**a**) and dye-added mixtures (i.e., with 0.01 wt % TMPyP) (**b**) with nitrogen inertization and at a light intensity of 12 mW cm^−2^, Figure S4: Molar percentages of α-HAP released into the PBS solution for the three photoinitiator formulations, Figure S5: Mass spectrum of 2-Hydroxy-1-[4-(2-hydroxyethoxy) phenyl]-2-methyl-1-propanone and possible cleavage products, Figure S6: Mass spectrum of the main cleavage product of (4-(2-Hydroxy)-ethoxybenzaldehyde), Figure S7: Mass spectrum of PEGDA 700 and its oligomers, Figure S8: Total ion chromatogram (orange) and selected ion chromatograms at different mass to charge ratios (black *m*/*z* = 121, red *m*/*z* = 99; blue *m*/*z* = 59, pink *m*/*z* = 55) of the washing solution after 24 h, Figure S9: Relative concentration of photoinitiator (red) and photo cleavage product (black) in the washing solutions, Table S1: Photopolymerization data including final conversions, induction times and maximum reaction rates for all samples measured by RT-FTIR spectroscopy (with and without TMPyP, with three different α-HAP concentrations and under air or inert atmosphere).
