# Supplementary material for: Transparent Low Molecular Weight Poly(Ethylene Glycol) Diacrylate-Based Hydrogels as Film Media for Photoswitchable Drugs
Source: Polymers (Basel). 2017 Nov 23;9(12):639. doi: 10.3390/polym9120639 (PMC6418822; doi:10.3390/polym9120639)
Supplement: Supplementary file 1 [file polymers-09-00639-s001.pdf]

Transparent Low Molecular Weight Poly(Ethylene Glycol) Diacrylate-Based Hydrogels as Film Media for Photoswitchable Drugs

Electronic supplementary information (ESI)

Théophile Pelras,[a,b,*] Sarah Glass,[a,*] Tom Scherzer[a], Christian Elsner,[a] Agnes Schulze,[a,†] and Bernd Abel[a]

a. Leibniz-Institute of Surface Modification, Permoserstraβe 15, 04318 Leipzig, Germany
b. Key Center for Polymers and Colloids, School of Chemistry, University of Sydney, NSW 2006, Australia
[*] Both authors contributed equally to this publication
† for correspondence: agnes.schulze@iom-leipzig.de

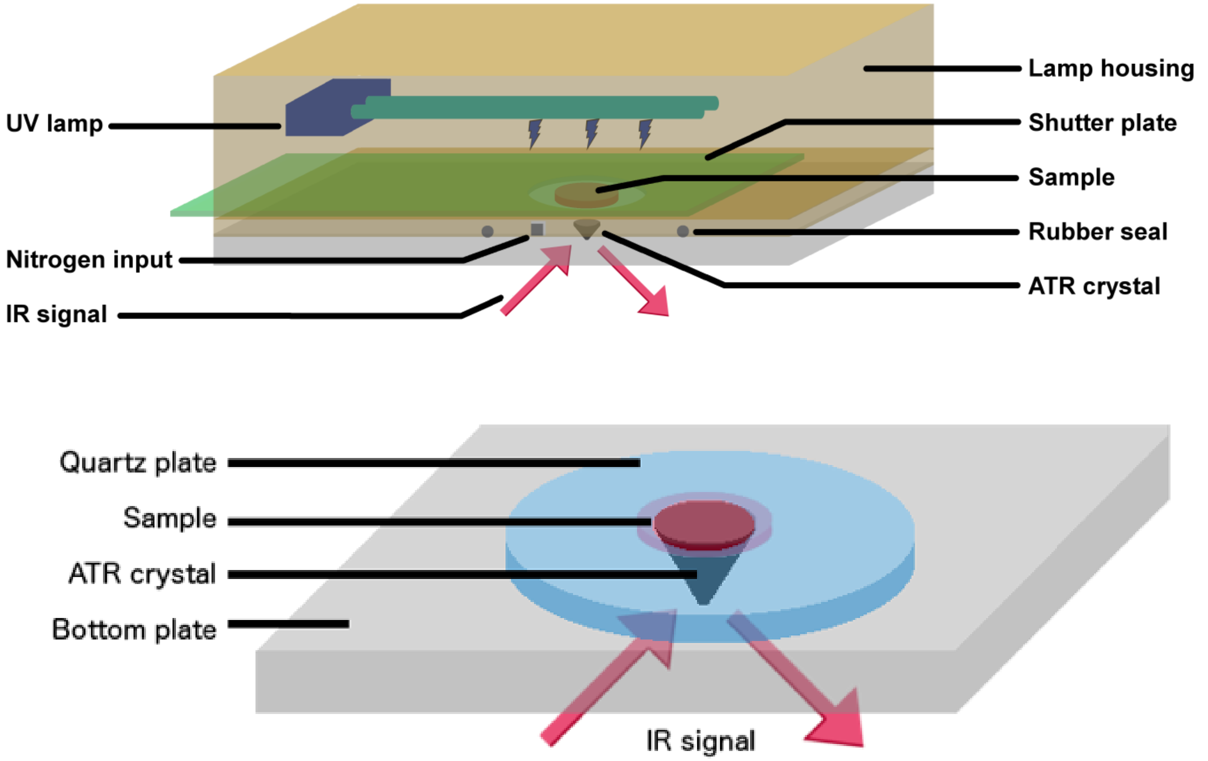

Figure S1: Scheme of the lamp and its irradiation box used for the photopolymerization of the mixtures (top). The scheme shows the irradiation box mounted on the ATR module of the RT-FTIR spectrometer. Detailed view of the sample deposited on the ATR crystal and calibrated using the quartz plate (bottom).

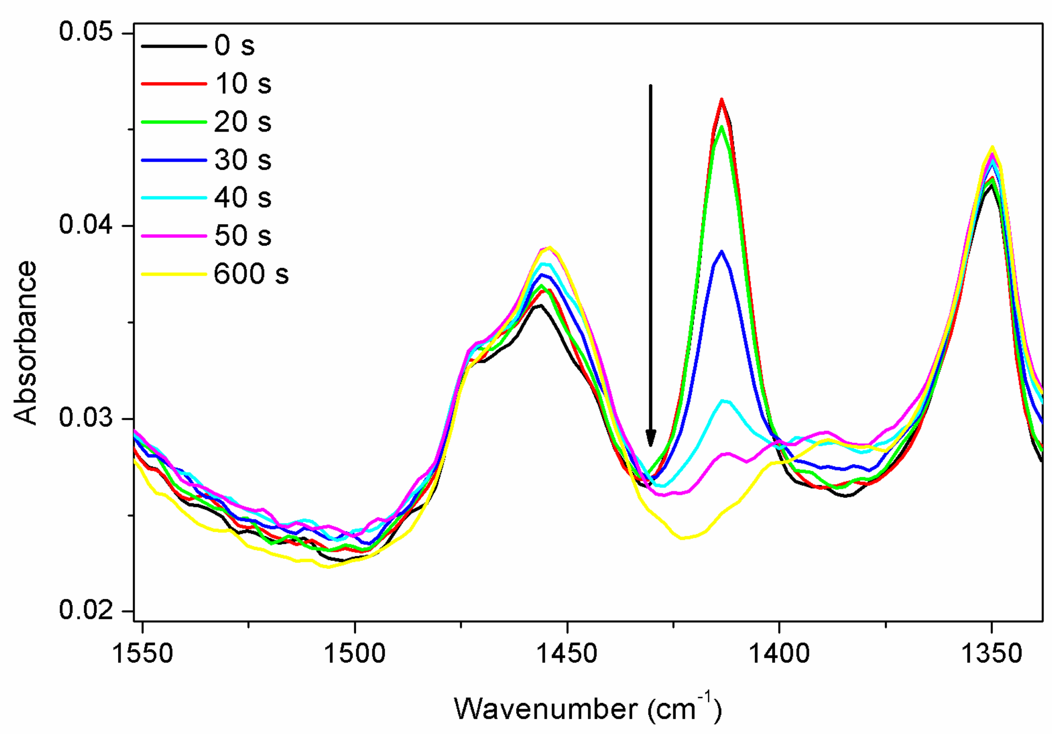

Figure S2: Decrease of the IR band assigned to the =CH2 deformation vibration of the PEGDA at 1,410 cm^{-1} in a hydrogel sample. The formulation containing 0.5 wt% α-HAP was photopolymerized in air with a light intensity of 12 mW cm^{-2} using the UV-A lamp.

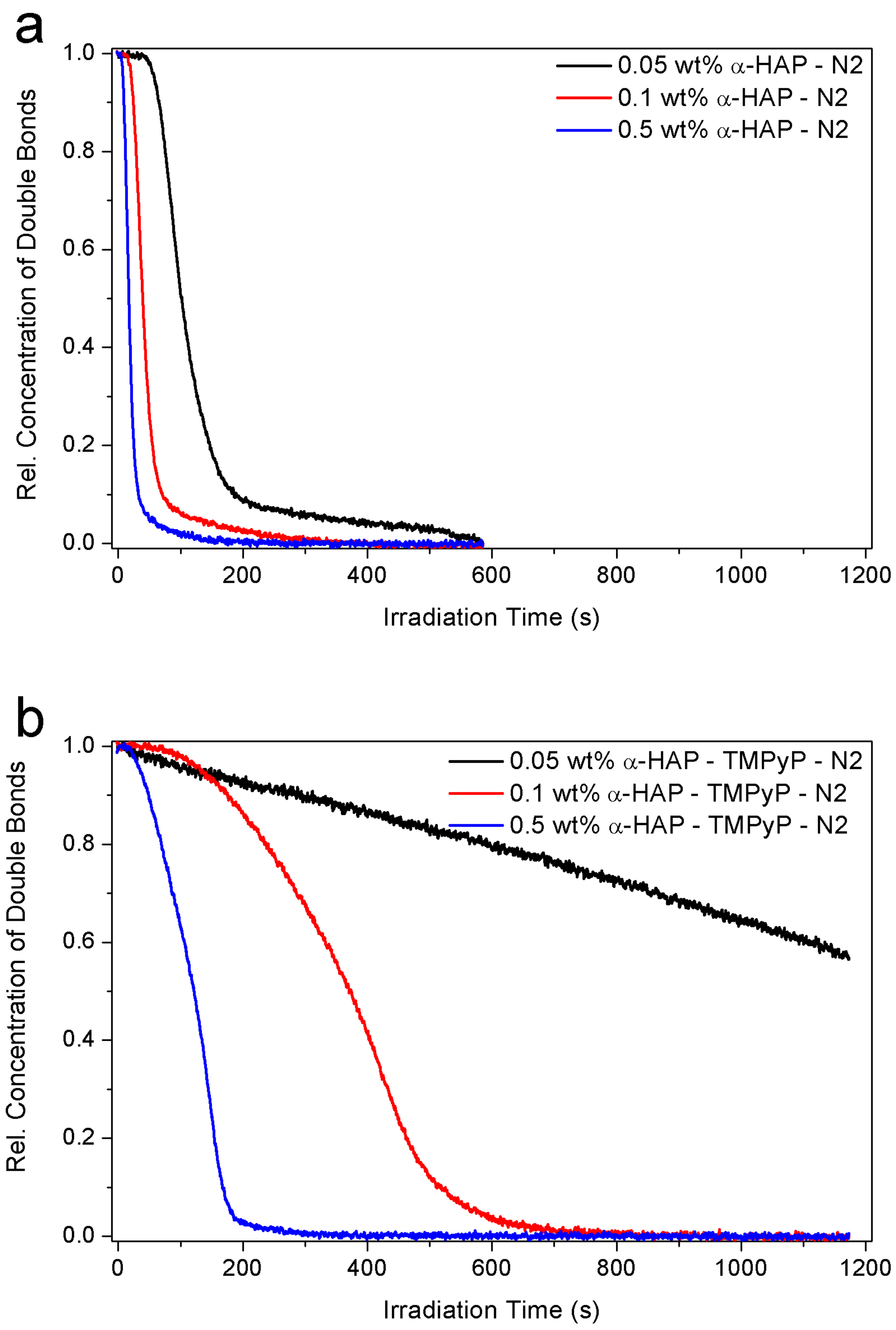

Figure S3: Photopolymerization kinetics of hydrogel formulations using three different α-HAP concentrations in dye-free formulations (up) and dye-added mixtures (i.e. with 0.01 wt% TMPyP) (down) with nitrogen inertization and at a light intensity of 12 mW cm^{-2}.

[α-HAP] (wt%)	0.05		0.05		0.1		0.1		0.5		0.5	
[TMPyP] (wt%)	0		0.01		0		0.01		0		0.01	
Atmosphere	air	N_2	air	N_2	air	N_2	air	N_2	air	N_2	air	N_2
Conv. at 300 s (%)	97	94	10	11	98	99	23	33	100	100	99	99
Conv. at 600 s (%)	100	100	18	21	100	100	78	96	100	100	100	100
Final conv. (%)	100	100	37	43	100	100	100	100	100	100	100	100
Induc. time (s)	35	50	65	65	20	15	55	100	5	5	19	26
Max. rate (s^{-1})	0.008	0.012	0.0004	0.0004	0.014	0.020	0.002	0.003	0.033	0.053	0.012	0.010

Table S1: Photopolymerization data including final conversions, induction times and maximum reaction rates for all samples measured by RT-FTIR spectroscopy (with and without TMPyP, with three different α-HAP concentrations and under air or inert atmosphere).

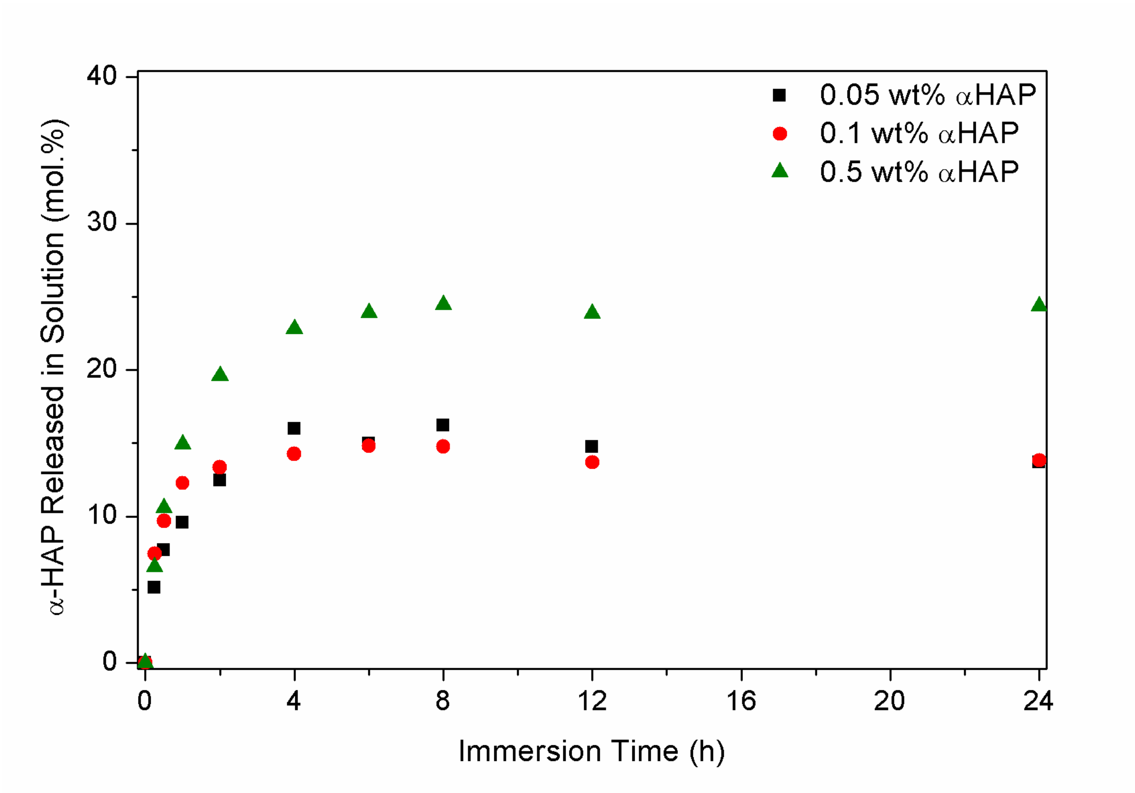

Figure S4: Molar percentages of α-HAP released into the PBS solution for the three photoinitiator formulations.

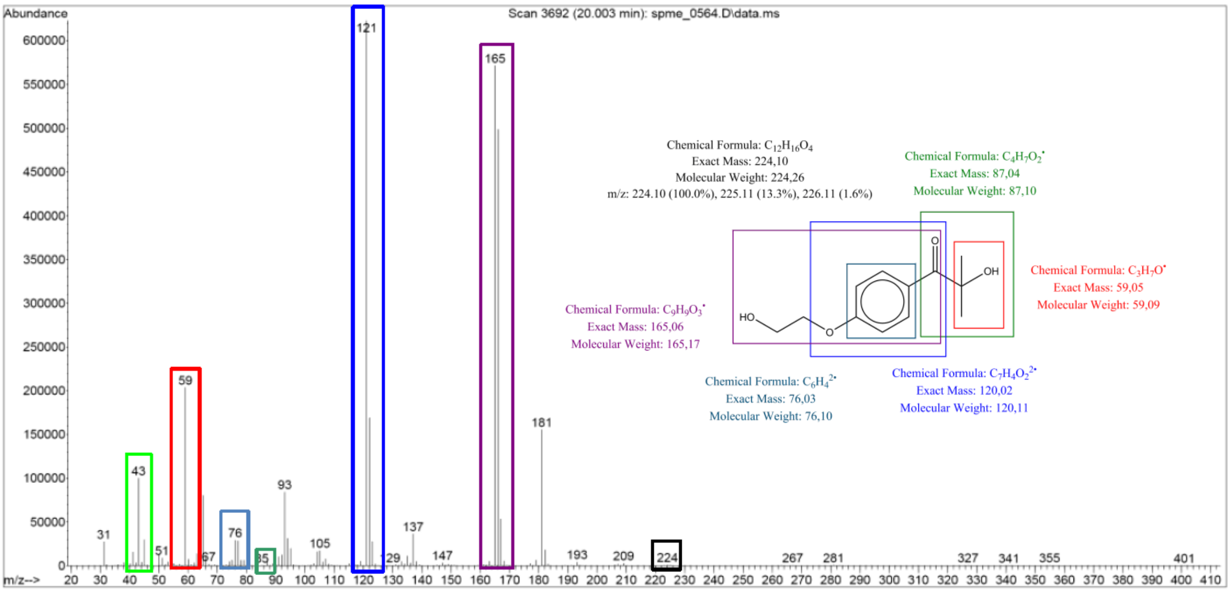

Figure S5: Mass spectrum of 2-Hydroxy-1-[4-(2-hydroxyethoxy) phenyl]-2-methyl-1-propanone and possible cleavage products.

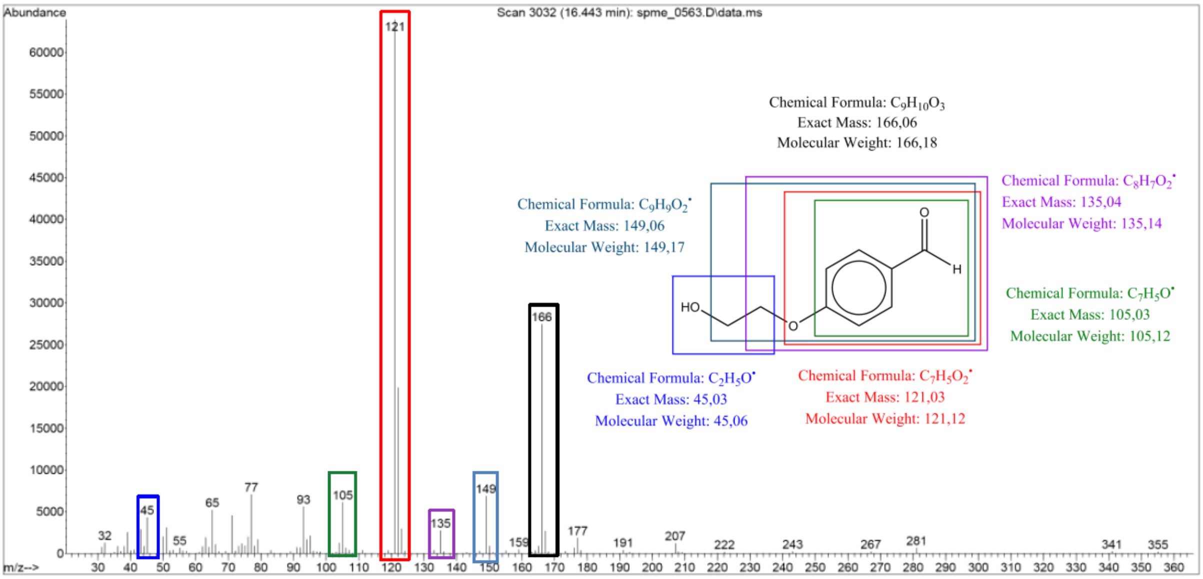

Figure S6: Mass spectrum of the main cleavage product of (4-(2-Hydroxy)-ethoxybenzaldehyde).

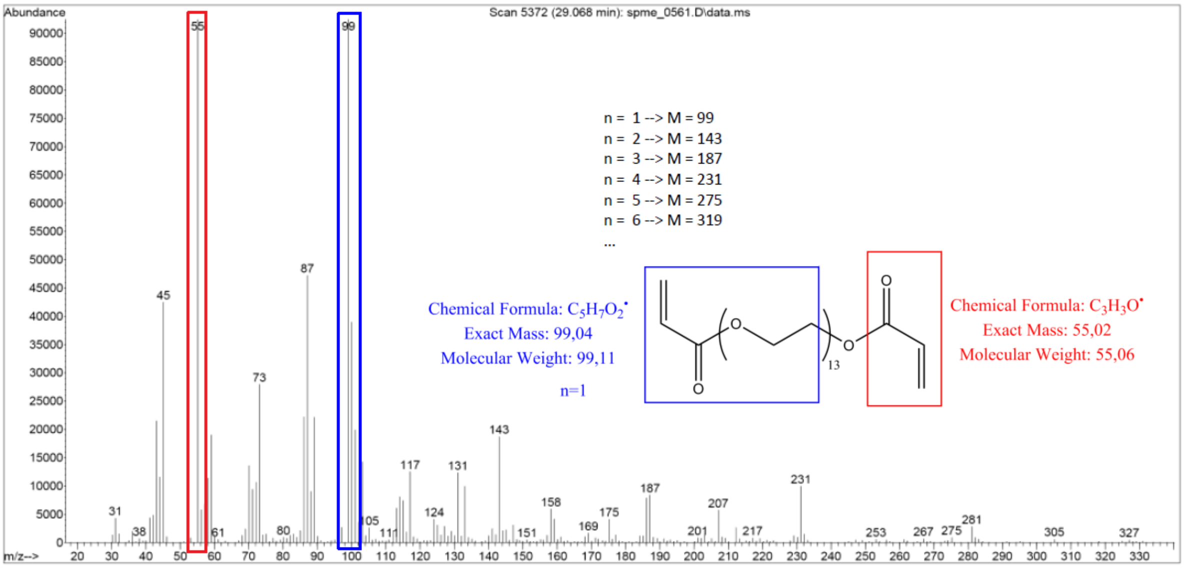

Figure S7: Mass spectrum of PEGDA 700 and its oligomers.

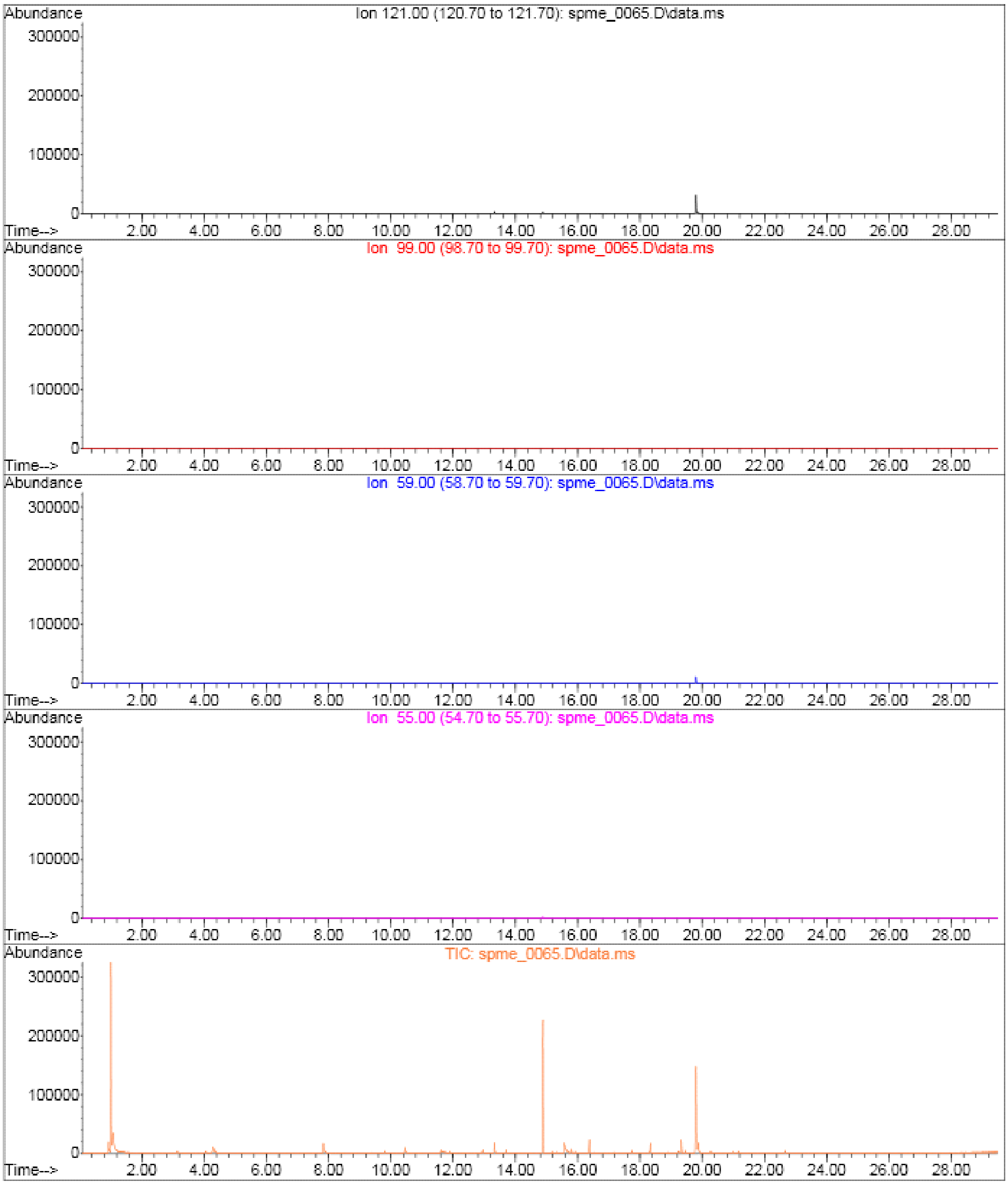

Figure S8: Total ion chromatogram (orange) and selected ion chromatograms at different mass to charge ratios (black m/z = 121, red m/z = 99; blue m/z = 59, pink m/z = 55) of the washing solution after 24 h.

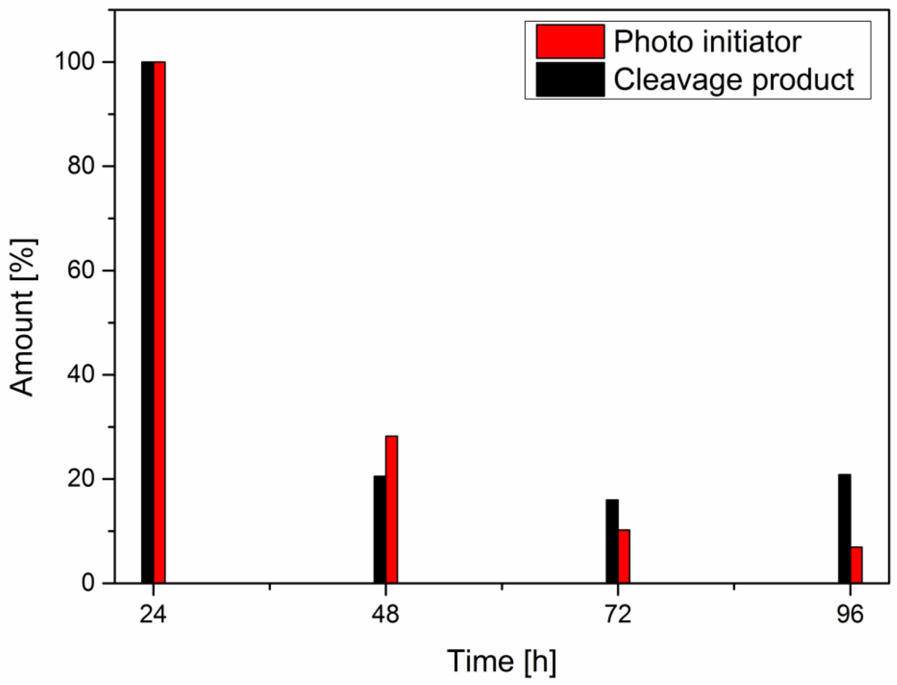

Figure S9: Relative concentration of photoinitiator (red) and photo cleavage product (black) in the washing solutions.
